# A Universal Live Cell Barcoding-Platform for Multiplexed Human Single Cell Analysis

**DOI:** 10.1038/s41598-018-28791-2

**Published:** 2018-07-17

**Authors:** Felix J. Hartmann, Erin F. Simonds, Sean C. Bendall

**Affiliations:** 1 Department of Pathology, School of Medicine, Stanford University, Palo Alto, CA USA; 2 Department of Neurology, UCSF, San Francisco, CA USA

## Abstract

Single-cell barcoding enables the combined processing and acquisition of multiple individual samples as one. This maximizes assay efficiency and eliminates technical variability in both sample preparation and analysis. Remaining challenges are the barcoding of live, unprocessed cells to increase downstream assay performance combined with the flexibility of the approach towards a broad range of cell types. To that end, we developed a novel antibody-based platform that allows the robust barcoding of live human cells for mass cytometry (CyTOF). By targeting both the MHC class I complex (beta-2-microglobulin) and a broadly expressed sodium-potassium ATPase-subunit (CD298) with platinum-conjugated antibodies, human immune cells, stem cells as well as tumor cells could be multiplexed in the same single-cell assay. In addition, we present a novel palladium-based covalent viability reagent compatible with this barcoding strategy. Altogether, this platform enables mass cytometry-based, live-cell barcoding across a multitude of human sample types and provides a scheme for multiplexed barcoding of human single-cell assays in general.

## Introduction

In recent years, the development of high-dimensional single-cell technologies such as mass cytometry (also termed cytometry by time-of-flight; CyTOF) have enabled unprecedented insights into many biological and clinical questions, spanning research in hematopoiesis^1,2^, stem cells^3^, cancer^4–6^, and autoimmunity^7–9^. Together with newly developed data analysis approaches (reviewed in refs^10–13^), mass cytometry and other single-cell analysis methodologies provide an ideal platform for explorative studies, which oftentimes involve large groups of samples with unknown cellular composition. In order to improve sample comparability and inter-assay reproducibility, much effort has been invested into the standardization and quality control of mass cytometry experiments. Changes in instrument sensitivity across different days or during extended acquisitions have been addressed by implementing a daily tuning procedure^14^ and through the simultaneous acquisition of bead standards^15^. To further minimize technical variance from experimental procedures or data analysis, multiple samples can be combined and processed in parallel as one single sample via cellular barcoding. For mass cytometry, individual samples are tagged with a unique combination of heavy-metal isotopes such that all cells of a sample are permanently labeled with their respective identifier^16,17^. These labeled samples can then be combined into one composite sample for simultaneous downstream experimental handling including antibody staining, washing, fixation, and acquisition. Following data acquisition, individual cells can be unmixed *in silico* and reassigned back to their initial samples via their unique barcode.

First mass cytometry-specific barcoding approaches have relied on labeling cells with heavy-metals via amine- or sulfhydryl-reactive chelating agents^16,17^. As these groups are most abundantly found within cells, as opposed to their surface, fixation and permeabilization are required, making these methods less suitable for barcoding before probing of fixation- or permeabilization-sensitive molecules or epitopes.

These issues can be overcome by making use of cell-surface molecules for barcoding purposes. For instance, the protein tyrosine phosphatase, receptor type C (CD45) has been proposed as a candidate antigen for live-cell barcoding using chelated palladium isotopes^18,19^. Still, the varied tissue expression pattern and weak palladium signal limit this approach in applicability to cells highly expressing CD45, namely peripheral blood mononuclear cells (PMBCs). Instead, we have devised a live-cell barcoding method robust to cell origin and identity. To do so, we targeted a combination of ubiquitously expressed cell surface molecules with cisplatin-conjugated antibodies^20^. We then demonstrate broad applicability of this approach in research involving human stem cells, immune cells as well as a broad range of different cancer cell lines and patient samples.

## Results

### MHC-I and sodium-potassium ATPase-subunits are broadly expressed across multiple human cell types

**Figure 1:**
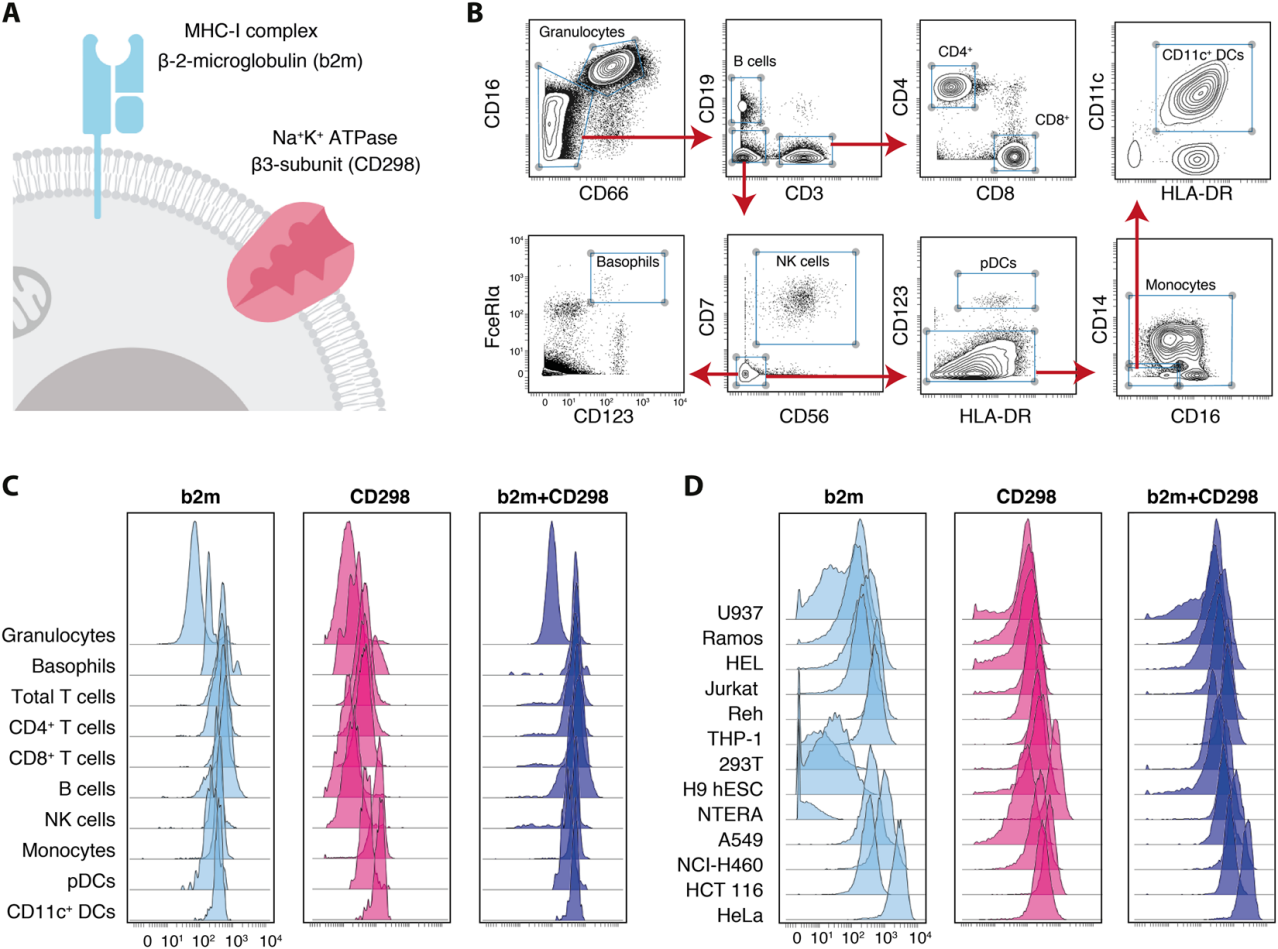
MHC-I and sodium-potassium ATPase-subunits are broadly expressed across different cell types. (**A**) The two surface proteins b2m as part of the MHC-I complex and CD298, a subunit of the sodium potassium pump, were selected as potential targets for live cell barcoding. (**B**) Human whole blood was subjected to red blood cell (RBC) lysis and subsequently stained with heavy-metal isotope-conjugated antibodies for mass cytometry (Table S1). Cells were pre-gated on live, single, CD45^+^ and CD235ab^-^. The main immune lineages were then identified via the indicated gating scheme. (**C**) Expression of b2m (light blue, left), CD298 (magenta, middle) and combined expression values from b2m and CD298 using the same reporter isotope (dark blue, right) on immune populations as in B. (**D**) Expression of b2m (light blue, left), CD298 (magenta, middle) and combined expression values from b2m and CD298 using the same reporter isotope (dark blue, right) on various cancer cell lines. Shown is one representative experiment out of two.

To facilitate robust barcoding of live human cells of different origin, we first identified cell surface proteins which were reported to be broadly expressed across different immune cell subsets, various organs^21^ and in cancer cell lines^22,23^. Further requirements were high epitope abundance as well as the availability of an antibody probe for robust detection of the target. Based on these criteria, we conjugated antibodies against beta-2-microglobulin (b2m) as part of the MHC class I complex as well as antibodies against the beta-3 subunit of the Na^+^/K^+^-ATPase (CD298) to heavy-metal isotopes for their use in mass cytometry (Fig. 1A). Next, we tested their expression on various cell populations, including immune cell subsets found in whole blood (Fig. 1B,C, see Table S1), as well as various cancer and non-immune cell lines such as leukemic (U937, Ramos, HEL, Jurkat, REH and THP-1), embryonic or stem cell-derived (293 T, H9 human embryonic stem cells (hESCs) and NTERA) and carcinoma cell lines (A549, NCI-H460, HCT 116 and HeLa; Fig. 1D). As expected, b2m was robustly expressed on all major immune cell subsets found in human whole blood. Granulocytes displayed slightly lower but still considerable expression of b2m. Most cancer cell lines also expressed b2m with the exception of low or absent expression levels on embryonic/stem cell lines and intermediate levels on a subset of leukemia cell lines (Fig. 1D; left).

CD298 was found to be expressed with intermediate levels on all identified immune cell subsets and with intermediate to high levels on the analyzed cell lines, including those which showed low to negative b2m staining (Fig. 1C,D; middle). Dying and/or dead cells might decrease surface expression of these molecules to varying extent (Fig. S1). Given these expression patterns, we reasoned that the combined staining of both molecules with antibodies conjugated to the same reporter isotopes would not only increase the number of available epitopes resulting in augmented staining intensity, but also increase its robustness and facilitate barcoding of heterogeneous samples containing multiple cell types. Indeed, combined staining of b2m and CD298 within the same detection channel demonstrated strong signal across all populations (Fig. 1C,D; right).

In summary, while the wide variety of cell types tested exhibited variability in their expression of b2m and CD298, there was no instance where the abundance of both was low at the same time, thus making their combination ideal for molecular barcoding.

### Monoisotopic cisplatin-conjugated antibodies are robust across analysis conditions

**Figure 2:**
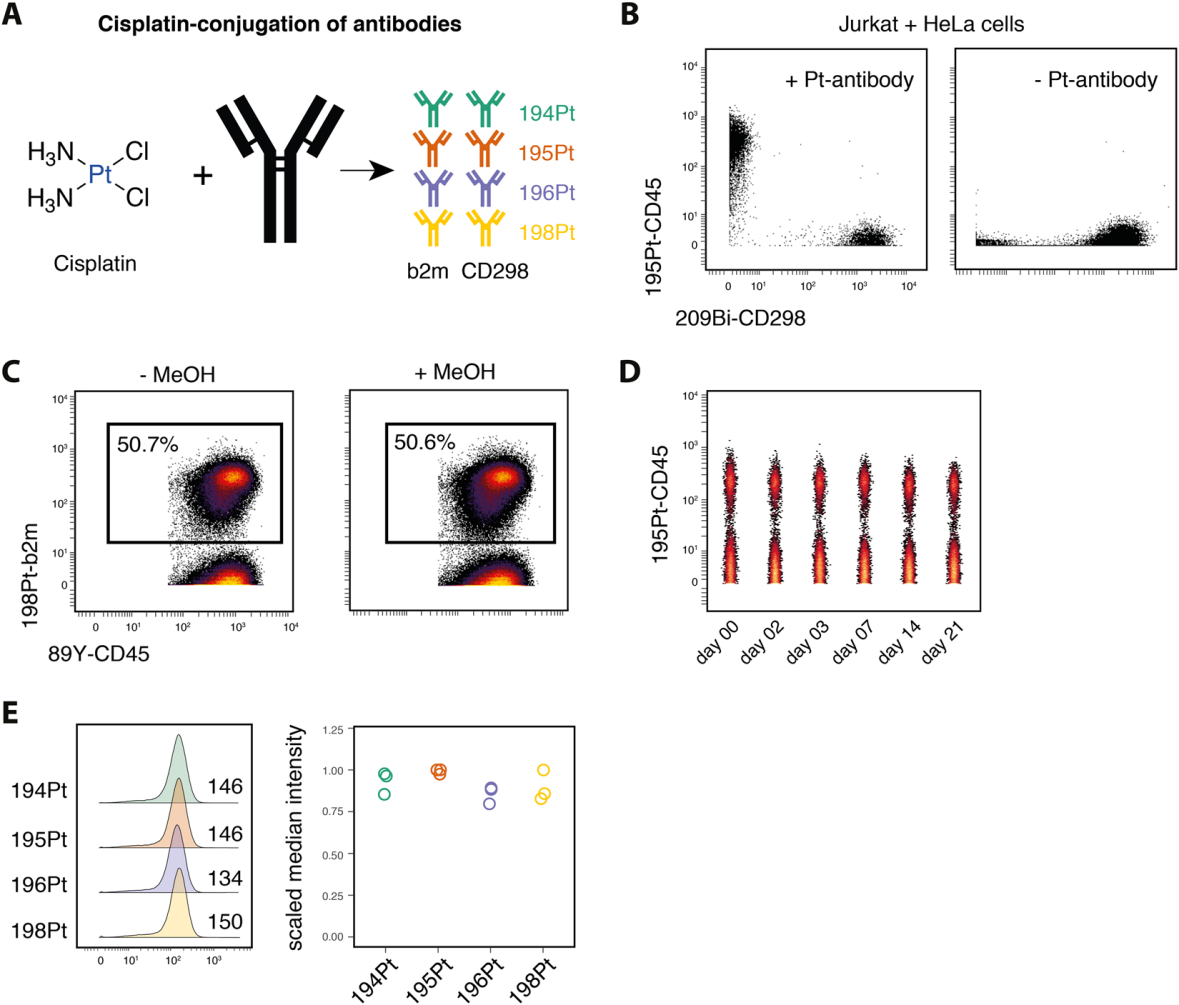
Cisplatin-conjugation of antibodies provides a specific and stable extension to lanthanide antibodies. (**A**) CD298 and b2m antibodies were conjugated to four different platinum isotopes (194Pt, 195Pt, 196Pt and 198Pt) via covalent binding of cisplatin. (**B**) Unstained Jurkat and 209Bi-stained HeLa cells were combined and stained with cisplatin-conjugated anti-CD45 (left) or no anti-CD45 antibody (right). (**C**) Cisplatin-antibody (anti-b2m) stained PBMCs and unstained PBMCs were mixed and acquired without (left) or after MeOH fixation (right). (**D**) Cells as in (**C**) were kept in intercalation solution at 4 °C for the indicated number of days before acquisition. (**E**) PBMC samples were stained with anti-b2m and anti-CD298 antibodies conjugated to different platinum isotopes. Numbers indicate median intensity in the respective channel (left). Median intensities were scaled to the maximum of the respective experiment (right).

To maximize the compatibility of this approach with existing mass cytometry assays, heavy-metal isotopes which are not part of and which do not interfere with the typically employed lanthanide range are desirable for barcoding. One possibility is the conjugation of EDTA-chelated palladium to antibodies^18^, which provided a first extension of the lanthanide range for antibody labeling. However, given the mass response curve of current mass cytometers, palladium isotopes suffer from a low detection sensitivity. Alternatively, cisplatin-based conjugation of platinum to antibodies has been reported to possess superior signal intensities compared to EDTA-chelated palladium antibodies^20^. Consequently, we conjugated anti-b2m and anti-CD298 antibodies to four different platinum isotopes (194Pt, 195Pt, 196Pt and 198Pt) via direct, covalent binding of cisplatin (Fig. 2A). Platinum-conjugated antibodies retained their initial specificity as shown by staining a mixture of CD45-positive (Jurkat) and CD45-negative (HeLa) cells with anti-CD45 antibodies. We did not detect any unspecific binding of platinum-conjugated anti-CD45 antibodies to CD45-negative HeLa cells (Fig. 2B), as had been occasionally observed with EDTA-chelated Pd antibodies (data not shown).

To assess the binding stability of cisplatin-antibodies to their target cells, cisplatin-antibody stained cells were fixed with 1.6% PFA and permeabilized using methanol (MeOH) before acquisition (Fig. 2C). Measured platinum-signal intensities from MeOH and non-permeabilized samples were comparable, indicating stable binding of platinum-antibodies to their target epitopes which is not majorly disrupted through standard cell processing methods. Further, we assessed storage stability of platinum-antibody stained samples at 4 °C in PFA-based intercalation solution (Fig. 2D). We observed a slight decrease signal intensity after prolonged sample storage, similar to what has been reported for lanthanide-conjugated antibodies^24^. Importantly, even after 21 days of storage at 4 °C, positive and negative populations could still be readily resolved. Lastly, to compare the sensitivity of the different platinum channels on our mass cytometer (CyTOF2), we stained samples with the same antibody clones conjugated to different platinum-isotopes (Fig. 2E). Measured signal intensities across the four platinum channels were found to be very similar, indicating that these channels have equivalent potential for use in mass cytometry applications, including barcoding. As such, monoisotopic cisplatin antibody conjugates provide a practical means for live-cell barcoding and do not interfere with lanthanide-based target protein measurements.

### Combining b2m and CD289 for live-cell barcoding of heterogeneous human samples

**Figure 3:**
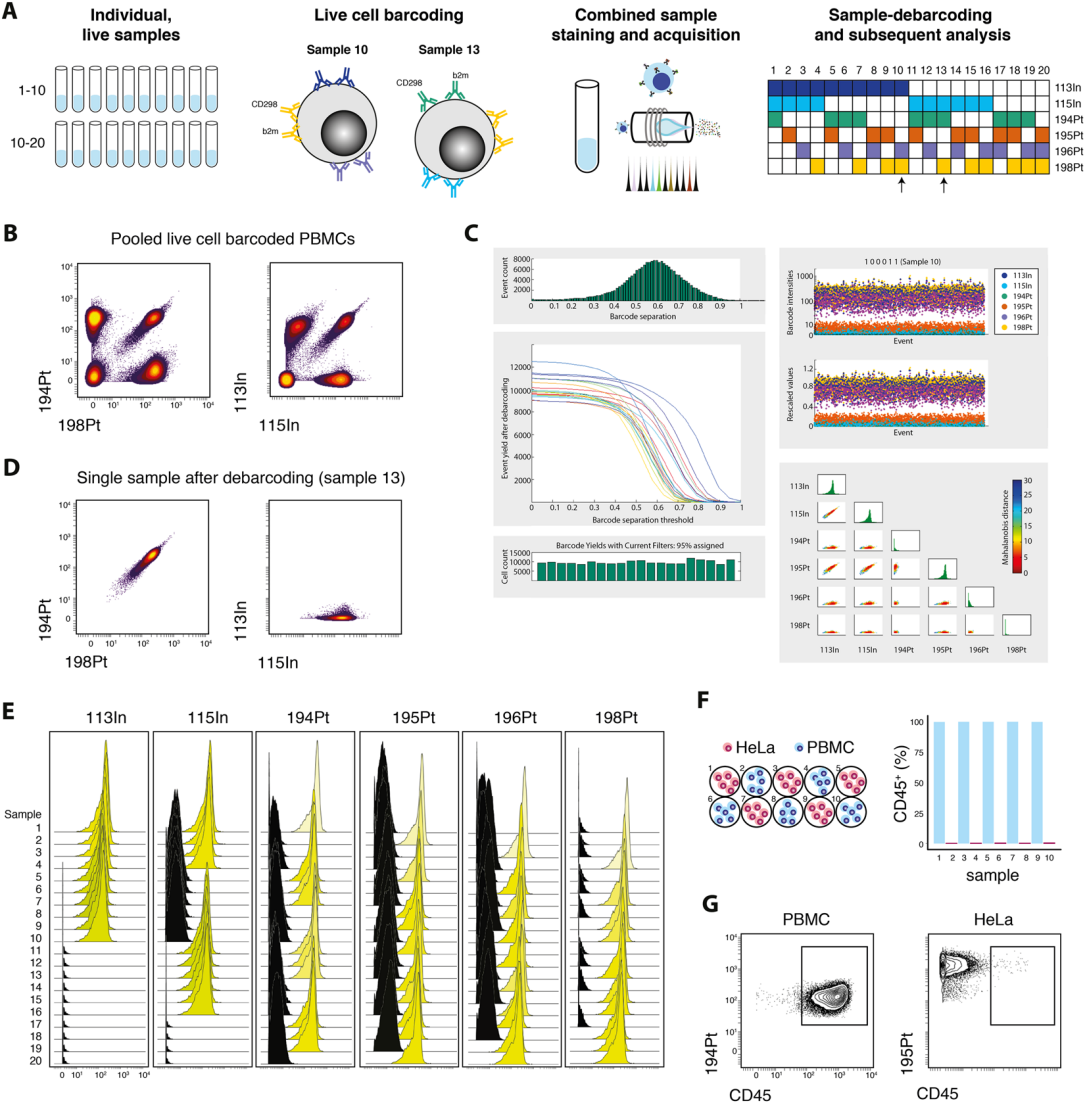
CD298 and b2m enable robust live cell barcoding of heterogeneous samples. (**A**) Schematic representation of the barcoding procedure. Individual samples are barcoded with a combination of three different b2m and CD298 antibodies, pooled, stained and acquired. Samples can then be debarcoded using the supplied barcoding matrix. Arrows point out sample 10 and 13 which are used as examples in A and C. (**B**) 20 PBMC samples were barcoded using the above described method. Shown are live, single cells from combined samples before debarcoding. (**C**) Combined samples can be debarcoded using existing algorithms and software. (**D**) Example of a debarcoded sample positive for three of the available six barcodes. (**E**) Signal intensities of all barcode isotopes from combined anti-b2m and anti-CD298 antibodies on debarcoded samples as in E. (**F**) PBMC and HeLa samples were barcoded, combined and stained with anti-CD45 (left). Frequency of CD45^+^ in debarcoded cells (right). (**G**) Exemplary biaxial plots demonstrating CD45 staining in debarcoded cells as in F, assigned to a PBMC (left) or HeLa sample (right).

Having identified suitable target epitopes and conjugation procedures, we combined these approaches to demonstrate their applicability to live cell barcoding. In order to extend the number of possible barcode combinations, we used indium isotope-conjugated (113In and 115In) anti-b2m and anti-CD298 antibodies in addition to four cisplatin-conjugated antibodies, as described above. Employing a 6-choose-3 scheme enables the barcoding of up to 20 samples with clear doublet identifying ability^17^. PMBCs from healthy donors were distributed into 20 individual samples and barcoded by staining the individual samples with combined anti-b2m and anti-CD298 in three out of the six available isotopes (Fig. 3A). Barcoded cells were combined, surface-stained, fixed and MeOH permeabilized before acquisition on a CyTOF2 mass cytometer. Barcode-positive and negative populations were readily distinguishable using both cisplatin and chelator-based conjugation methods (Fig. 3B). The combined sample was then loaded into existing matlab-based debarcoding software^17^ to automatically assign cells back to their initial identity (Fig. 3C). Up to 95% of input cells were assigned to their respective samples, using appropriate debarcoding parameters (cutoff: 0.1; Mahalanobis distance: 30). Debarcoded samples displayed the expected staining patterns according to their assigned barcode (Fig. 3D–E). Next, we tested the reliability and feasibility of barcoding different sample types, including non-immune cell subsets, into one reaction vessel. We prepared individual samples from human PBMCs or human epithelial HeLa cells, performed live cell barcoding, combined all samples into one vessel and subsequently stained for CD45 surface expression (Fig. 3F,G). To test the overall performance after acquisition and debarcoding we assessed the expression of CD45, a marker expected to be ubiquitously expressed by PBMCs but not HeLa cells. Virtually all cells (>99.5%) assigned to PBMC samples were found to express CD45 and vice versa, HeLa samples were not found to contain CD45^+^ cells (Fig. 3G). Together these data demonstrate that the combination of anti-b2m and anti-CD298 antibodies enables highly multiplexed, doublet-free live-cell barcoding of a variety of human cell types.

### A novel covalent palladium-based viability staining

**Figure 4:**
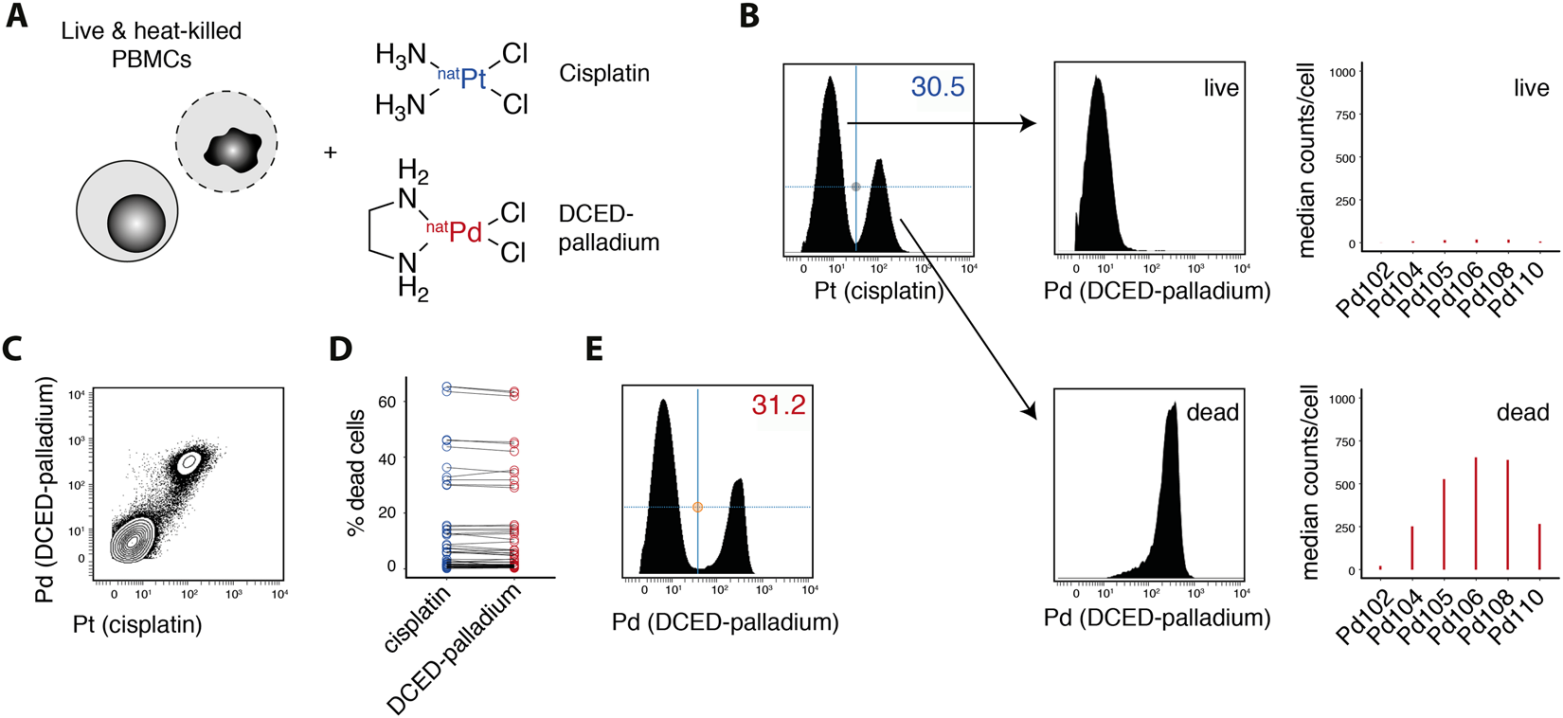
Palladium-based viability staining reproduces cisplatin-based dead cell identification. (**A**) Live and heat-killed (55 °C for 1 h) PBMCs were mixed and incubated for 5 min at RT with 500 nM cis-dichloro-diamine-platinum(II) (cisplatin) and 500 nM dichloro-(ethylenediamine)-palladium(II) (DCED-palladium). (**B**) Platinum (198Pt) signal (left) in cells as in (**A**) was used to discriminate live (cisplatin^low^) and dead cells (cisplatin^high^), which were then analyzed for their palladium signal in one palladium channel (110 Pd, middle) or across all measured palladium isotopes (right). 110 Pd is shown as an example but other Pd-channels (with the exception of 102 Pd) could be used analogously. Representative example from a total of n = 8. (**C**) DCED-palladium (110 Pd) and cisplatin (198Pt) in cells as in (**A**). (**D**) The frequency of dead cells within multiple different types of samples was assessed using cisplatin and DCED-palladium simultaneously (n = 48, pooled from 10 independent experiments). (**E**) DCED-palladium on its own can be used to discriminate live from dead cells (here heat-killed as in A). Representative example from a total of n = 48.

Platinum, via cisplatin staining, is often used as a viability probe in mass cytometry because of its preferential accumulation in membrane-compromised cells and its covalent binding properties^25^. As our proposed barcoding strategy (Fig. 3) makes use of all currently available purified platinum isotopes for antibody labeling, we examined potential alternatives for covalent viability probes. Given its structural similarity to cisplatin, we investigated the use of dichloro-(ethylenediamine) palladium(II) (termed ‘DCED-palladium’) to identify membrane-compromised (dead) cells (Fig. 4A). Mixtures of live and heat-killed PBMCs were stained simultaneously with cisplatin and DCED-palladium for 5 min at RT (Fig. 4A). Dead cells were readily identifiable by their increased binding of cisplatin compared to live cells (Fig. 4B). Further, while cisplatin^low^ cells showed negative to low signal for DCED-palladium, we found cisplatin^high^ cells to have strong signal in the indicated palladium channels, following the naturally occurring abundance of the different isotopes (Fig. 4B). This correlation between cisplatin and DCED-palladium binding is further confirmed by directly analyzing platinum and palladium signal in a co-stained, live and heat-killed cell mixture (Fig. 4C). Again, dead cells were found to be positive for cisplatin as well as DCED-palladium.

Lastly, we tested whether the correlation between platinum-positive and palladium-positive frequencies holds true in various biological sample types. We routinely co-stained multiple samples including, PBMCs, digested tumor- and healthy-tissue samples and cancer cell lines with both viability probes. These samples spanned a wide range of dead-cell frequencies and furthermore, contained samples which were permeabilized for intracellular staining steps with either MeOH-based protocols or commercially available transcription factor staining kits. Importantly, DCED-palladium provided highly comparable dead-cell identification across all sample types as well as the full range of dead-cell frequencies (Fig. 4D), thus indicating that it can be readily used on its own to replace cisplatin as a viability probe and thus allow the full use of platinum-conjugated antibodies in mass cytometry experiments (Fig. 4E).

### Live cell barcoding enables the interrogation of tumor-immune composition and function

**Figure 5:**
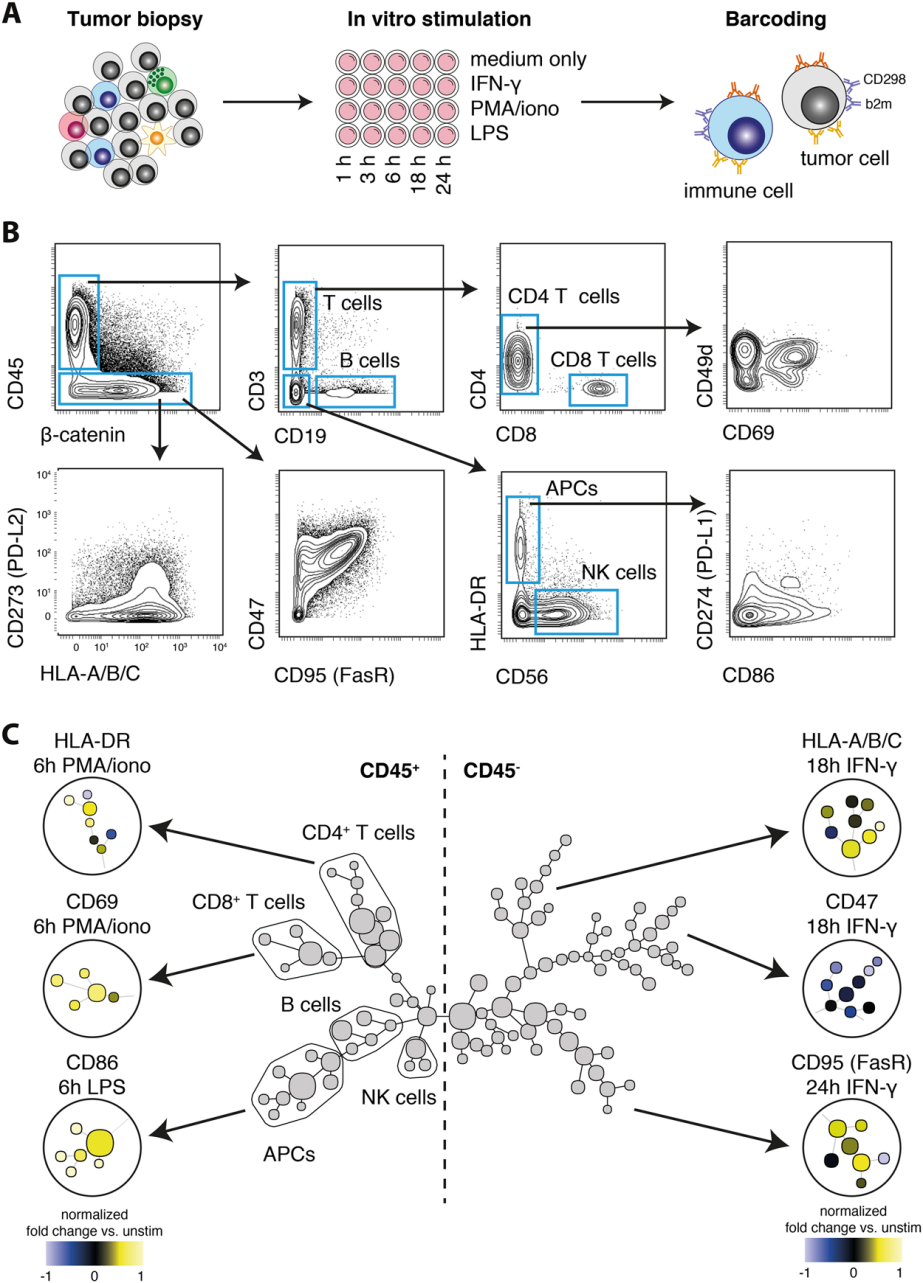
Live cell barcoding enables the combined investigation of tumor- and immune cell phenotypes and interplays. (**A**) Single-cell suspensions were prepared from a lung tumor tissue biopsy and stimulated *in vitro* for different periods of time with the indicated reagents. Tumor as well as immune cells from individual conditions were then live cell barcoded, combined and stained for immune-relevant markers (Table S1). (**B**) Different immune as well as tumor cell populations were identified via the indicated gating scheme and analyzed for their expression of multiple surface markers. APCs = antigen presenting cells. (**C**) Combined data was subjected to SPADE clustering (100 clusters) and represented as a minimal spanning tree (MST). Selected clusters are overlaid with a color-scheme representing the normalized fold-change in unstimulated vs. the indicated stimulus.

Lastly, we employed the above-described methodology (Fig. 3 and 4) to perform live cell barcoding of an exemplary, clinically relevant sample: a tissue biopsy of a lung carcinoma containing a heterogeneous mixture immune and non-immune cells (Fig. 5A). Firstly, the tissue sample was digested to prepare a single-cell suspension. We independently assessed the impact of enzymatic digestion on cellular b2m and CD298 surface expression and found that, under these conditions, cells retained sufficiently high surface expression for robust live cell barcoding (Fig. S2). Next, the sample was divided and activated for varying time periods (1–24 h) with different stimuli including interferon-γ (IFN-γ), lipopolysaccharide (LPS) or phorbol 12-myristate 13-acetate (PMA) and ionomycin, resulting in a total of 20 different conditions. Differentially stimulated samples were live-cell barcoded, combined and stained with heavy-metal conjugated antibodies (Table S1). After acquisition and debarcoding, multiple immune cell subsets but also non-hematologic cells could be identified and directly interrogated for their expression of immunologically important surface markers (Fig. 5B).

Additionally, we automatically identified different cell lineages via the SPADE algorithm^26^ (see also Fig. S3), giving an immediate overview of stimulation-induced changes in surface protein expression on immune but also on tumor cells. Fold changes of protein expression induced by the indicated stimuli were overlaid on selected parts of the tree structure (Fig. 5C).

Using our methodology, we confirmed the well-described upregulation of the activation-associated proteins HLA-DR or CD69 by PMA/ionomycin on various T cell subsets, as well as LPS-induced augmented expression of the co-stimulatory protein CD86 on antigen-presenting cells (APCs). Importantly, in addition to the changes detected in immune cells, interesting observations could be made on tumor cells simultaneously, including well-described IFN-γ induced upregulation of HLA class I expression^27^, downregulation of the anti-phagocytic protein CD47 [ref.^28^] or upregulation of the programmed cell death receptor CD95, also termed FasR [ref.^29^] on these cells (Fig. 5C). Together, this demonstrates the utility of the platform we have presented here to interrogate heterogeneous samples such as primary human tumor and immune cell mixtures to achieve a more integrated view of potential cellular interactions in a setting where the limitation of technical variability is crucial.

## Discussion

Cellular barcoding approaches have been proposed for a variety of technologies, including single-cell sequencing^30^, antibody/sequencing hybrid technologies^31^, genetic barcoding for cell lineage tracing^32^, fluorescent barcoding for flow cytometry^33^ and more recently heavy-metal isotope based barcoding in mass cytometry^17–19,34^.

In contrast to many previous methods, our approach does not require samples to be fixed or permeabilized prior to barcoding and surface staining and is thus especially suited for scenarios in which target molecules or downstream assays are fixation- or detergent-sensitive or in cases in which this is unknown. Further, the above-described method is independent of CD45 expression which has been proposed for live cell barcoding of PBMCs before^18,19^. This now allows barcoding of cells of non-immune origin, including tumor cells or stem cell populations. Additionally, within leukocytes, CD45 expression is variable and informative for cell lineage identification. Our CD45-independent barcoding therefore circumvents elaborate methods involving pre-barcode, non-saturating surface staining of CD45 [ref.^35^] and enables easy incorporation of CD45 into the analysis panel.

Instead of CD45, we here made use of the broad expression of b2m as part of the MHC-I complex and the CD298 subunit of the sodium-potassium ATPase. Both proteins have been shown to be broadly expressed across different types of samples and biclonal barcoding using these two targets provides increased signal intensities and robustness against potential downregulation, e.g. on tumor cells^36^. Further, using this combination allowed us to confidently assign up to 95% of cells to individual samples, which is similar to or exceeding previously reported frequencies^17^.

Homologous protein-complexes of the human MHC-I and sodium-potassium ATPases are also expressed by many commonly used model organisms including mice and rats and thus, this method should be readily transferable to research questions in other species. Additionally, staining with a combination of anti-human b2m and CD298 antibodies could also serve to robustly identify human cell in various xenograft models^23^.

As aforementioned, ideal elemental isotopes for barcoding are outside of the lanthanide range typically reserved for the analysis panel. Therefore, we here employed four different platinum isotopes, 194Pt, 195Pt, 196Pt and 198Pt. Besides these, 190Pt and 192Pt occur naturally but with very low abundance and were therefore not available in sufficient enrichment grades, however they could become obtainable in the future. Platinum isotopes were conjugated to antibodies via direct binding of cisplatin which has been found to covalently react with cysteine residues^37^. Thus, conjugation does not require additional chelating-agents and chelating reactions, making the labeling reaction economical, rapid and uncomplicated. Antibodies are labelled analogously to familiar protocols and no additional equipment is needed. Furthermore, the platinum-based barcoding ensures that there is no interference through isotopic impurity or oxide-formation with the lanthanide range, thus minimizing interaction with the analytical channels for single cell quantification. At the same time, it should be noted that for individuals which have received platinum-based therapeutic agents (i.e. carboplatin or cisplatin), the cellular background of platinum should be assessed beforehand.

Since cisplatin is often employed as a live/dead probe in mass cytometry, alternative approaches have to be considered when using all four commonly available platinum isotopes for antibody-based staining. The use of rhodium (103Rh) intercalator to label dead cells has been reported before^38^, however due to its non-covalent binding, signal-intensity is lost in downstream fixation/permeabilization and wash steps making its application less robust. Additionally, amine- and thiol-reactive derivatives of EDTA or 1,4,7,10-tetraaza-cyclododecane-1,4,7,10-tetraacetic acid (DOTA) chelators have been used with non-biological metals to identify dead cells^39,40^. However, these reagents are expensive, chemically unstable, and require pre-formulation with the metal of interest. Instead, the here presented DCED-palladium provides a straightforward identification of dead cells in mass cytometry experiments. DCED-palladium is inexpensive, easy to use and store, and provides good separation between live and dead cell populations. Similar to cisplatin, it is compatible with downstream sample permeabilization and washing steps. DCED-palladium therefore provides an attractive alternative to cisplatin as a viability reagent, even in scenarios where cisplatin antibodies are not used for barcoding but instead to extend the analysis panel beyond the lanthanide range^20^. If needed, monoisotopic DCED-palladium could be synthesized in a relatively easy procedure^41^. Importantly, as with many reagents used in mass cytometry, DCED-palladium should be handled with care so that oral and dermal contact as well as inhalation is prevented.

Using only indium and platinum isotope-conjugated antibodies and employing the described 6-choose-3 scheme described here, up to 20 samples can be barcoded and processed as one composite sample. However, it is easily possible to increase the maximum number of samples by including anti-CD298 and anti-b2m antibodies conjugated to other heavy metals, e.g. 89Y or 209Bi. Extending to such an 8-choose-4 scheme, up to 70 samples could be barcoded using this approach. As b2m and CD298 are expressed at high copy numbers per cell, the maximum number of multiplexing channels is virtually unlimited if one is willing to incorporate additional reporter metals.

As we have shown, the here described platform for live cell barcoding is especially useful for the analysis of samples containing heterogeneous populations, such as mixtures of tumor cells and tumor-infiltrating leukocytes. Given the recent success of immunotherapies using checkpoint inhibitors or CAR T cells, a huge number of clinical studies is currently underway and mass cytometry is poised to be employed to identify outcome-associated cellular signatures, follow disease progression and predict therapeutic responses^42^. Especially in such settings with large sample numbers in which the reduction of technical variability is critical, our approach should provide significant benefit and we thus hope that this study will contribute to further implement mass cytometry in clinical research.

## Methods

### Samples

All human samples were obtained and experimental procedures were carried out in accordance with the guidelines of the Stanford Institutional Review Board (IRB). Specifically, fresh whole human blood in heparin collection tubes or leukoreduction system (LSR) chamber contents (Terumo BCT) were obtained from the Stanford Blood Center. Tissue specimens were obtained from the Stanford Tissue Bank. These samples are generally byproducts of blood donation procedures and normal surgical pathology workflows, the collection of which is governed independently by the Stanford IRB. Written informed consent was obtained from all donors. The experimental procedures/protocols in combination with the samples used in this study were deemed human subject research exempt under Stanford IRB protocol #42195 which was reviewed by Stanford IRB panel IRB-98.

PBMCs were isolated via Ficoll (GE Healthcare) density gradient centrifugation and cryopreserved in fetal bovine serum (FBS, Omega Scientific) supplemented with 10% DMSO (Sigma). After freezing, samples were stored in liquid nitrogen. For tissue, single-cell suspensions were prepared using the MACS tumor dissociation kit (Miltenyi Biotec) according to the supplier’s recommendations. For experiments, cells were thawed by dropwise addition of pre-warmed RPMI-1640 (life technologies), supplemented with 10% FBS and 1x L-glutamine (Thermo Fisher) and washed twice. Whole blood was subjected to RBC lysis using 1x RBC lysis buffer (BioLegend), following the manufacturer’s instructions. Cell numbers were determined using an automated cell counter (TC20, BioRad).

### Cell Culture

Jurkat, U937, Ramos, HEL, Reh, THP-1 and NCI-H460 cells were cultured in RPMI-1640 supplemented with 10% FBS, 1x L-glutamine and 1x penicillin/streptomycin (Thermo Fisher). HeLa and 293 T cells were cultured in DMEM (Corning) supplemented with 10% FBS and 1x penicillin/streptomycin. NTERA cells were cultured in DMEM supplemented with 20% FBS and 1x penicillin/streptomycin. A549 were cultured in F-12K Medium (Thermo Fisher) and HCT 116 cells in McCoy’s 5a Medium (Thermo Fisher), both supplemented with 10% FBS, 1x L-glutamine and 1x penicillin/streptomycin. H9 hESCs were cultured feeder-free in StemMACS iPS Brew (Miltenyi Biotec) on a Matrigel Membrane Matrix (Thermo Fisher). All cell lines were cultured at 37 °C, 5% CO_2_. For sample collection, adherent cell lines were washed with PBS, dissociated by adding Accumax (Thermo Fisher) for 5 min at 37 °C and subsequently washed in their respective medium. Cells from cancer cell lines were fixed with 1.6% PFA in PBS (Electron Microscopy Sciences) for 10 min at RT and stored at −80 °C.

### Heavy-metal conjugation of antibodies

Conjugation of anti-human antibodies to heavy metal isotopes of the lanthanide series and indium was conducted using the MaxPar X8 antibody-labelling kit (Fluidigm) following the manufacturers recommendations. Where available, pre-conjugated antibodies were obtained from Fluidigm. Cisplatin-conjugation of antibodies was based on a previous report^20^. In short, 1 mM stock solutions of isotopically enriched cisplatin in DMSO (custom order, Fluidigm) was pre-conditioned for 48 h at 37 °C [ref.^43^] and afterwards stored at −20 °C. Antibody buffer exchange was performed by adding 100 μg anti-human b2m (clone 2M2) or anti-CD298 (clone LNH-94, both BioLegend) to a 50 kDa MWCO microfilter (Milipore) and centrifuging for 10 min, 12,000 g at RT, followed by a second wash with R buffer (Fluidigm). Antibodies were then reduced with 4 mM TCEP (Thermo Fisher) for 30 min at 37 °C and washed two times with C buffer (Fluidigm). 20 μl of the 1 mM monoisotopic cisplatin solution (equivalent to 20 nmol) was diluted in 1 ml of C buffer and added to the antibody in a 1.6 ml tube for 1 h at 37 °C for conjugation. Cisplatin-conjugated antibodies are then washed four times with 400 μl W buffer (Fluidigm) and the antibody is collected by two centrifugations (2 min, 1,000 g, RT) with 50 μl of W buffer with an inverted column into a new 1.6 ml collection tube. Protein content was assessed by NanoDrop (Thermo Fisher) measurement, antibody stabilization buffer (Candor Bioscience) was added to a final volume of at least 50 v/v % and antibodies were stored at 4 °C.

### Live cell barcoding

Individual samples of up to 3 × 10^6^ cells each were stained with combinations of platinum or indium-conjugated anti-b2m (1 µg/ml) and anti-CD298 (2 µg/ml) in cell staining medium (CSM: PBS with 0.5% BSA and 0.02% sodium azide (all Sigma)) for 30 min at RT. As described previously^17^, a 6-choose-3 scheme was used to ensure doublet identification and removal. Cells were washed twice with CSM and combined into a single reaction vessel for downstream staining and acquisition.

### Viability staining

DCED-palladium or cisplatin (both Sigma) was resuspended in DMSO, pre-conditioned for 48 h at 37 °C and stored at −20 °C. Pre-dilutions were made in PBS and stored at 4 °C for up to two weeks. Viability staining was performed by resuspending the sample in 1 ml of PBS and adding DCED-palladium to a final concentration of 500 nM, followed by incubation for 5 min at RT and washing with CSM.

### Staining procedure

All surface staining was performed in CSM for 30 min at RT. Where indicated, cells were fixed with 1.6% PFA in PBS for 10 min at RT and permeabilized with MeOH for 10 min on ice. Before acquisition, samples were resuspended in intercalation solution (1.6% PFA in PBS, 0.02% saponin (Sigma) and 0.5 μM iridium-intercalator or 0.5 μM rhodium-intercalator (both Fluidigm)) for 1 h at RT or overnight at 4 °C.

### Data acquisition

Before acquisition, samples were washed once in CSM and twice in ddH_2_O. All samples were filtered through a cell strainer (Falcon) and resuspended at 1 × 10^6^ cells/mL in ddH_2_O supplemented with 1x EQ four element calibration beads (Fluidigm) and finally acquired on a CyTOF2 mass cytometer (Fluidigm). Barcoded samples were acquired using the Super Sampler injection system (Victorian Airship).

### Data pre-processing and debarcoding

Acquired samples were bead-normalized using matlab-based software as described previously^15^. Normalized data was then uploaded onto the Cytobank analysis platform^44^ to perform initial gating on single, live cells based on their DNA content, their event_length parameter and the live/dead probe. Data was transformed with an inverse hyperbolic sine (arcsinh) transformation with a cofactor of 5. Where applicable, barcoded data was debarcoded using matlab-based software with the reported parameters^17^. Debarcoding parameters were adjusted to match the barcoding separation of the respective experiment.

### Data analysis and figure preparation

Basic gating and expression analysis was performed using the Cytobank environment. SPADE representations were created in Cytobank using the available implementation. Further downstream analysis was performed within the R software package^45^. Figures were prepared with biorender software and Illustrator (Adobe).

## Electronic supplementary material

Supplementary Figures

Table S1
