# Supplementary material for: A Universal Live Cell Barcoding-Platform for Multiplexed Human Single Cell Analysis
Source: Sci Rep. 2018 Jul 17;8:10770. doi: 10.1038/s41598-018-28791-2 (PMC6050312; doi:10.1038/s41598-018-28791-2)
Supplement: Supplementary file 1 — Supplementary Figures [file 41598_2018_28791_MOESM1_ESM.pdf]

Supplementary Information for:

A Universal Live Cell Barcoding-Platform for Multiplexed Human Single Cell Analysis

Felix J. Hartmann[1], Erin F. Simonds[2], Sean C. Bendall[1,*]

Fig. S1. Reduced barcoding target expression on dead cells.

Fig. S2. Influence of digestive enzymes on b2m and CD298 expression.

Fig. S3. SPADE representation of a tumor biopsy.

Table S1. Antibody panels related to Fig. 1 and Fig. 5

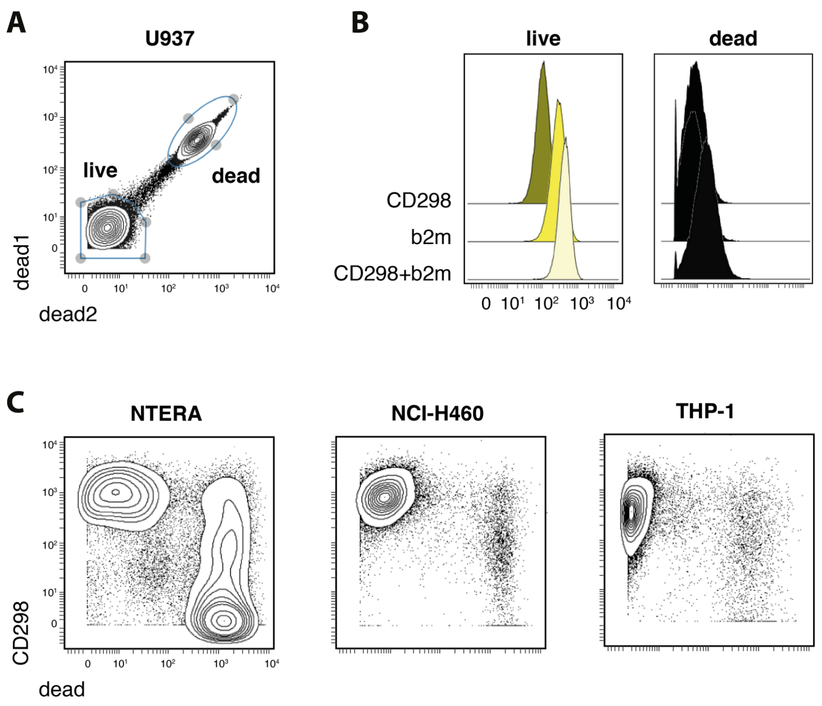

Fig. S1. Reduced barcoding target expression on dead cells. (A) U937 cells were stained with a live dead probe and analyzed by mass cytometry. Dead (membrane-compromised) cells can be easily separated from live (intact) cells. (B) CD298, b2m and combined expression of CD298 and b2m on live (left) and dead (right) cells gated as in A. (C) CD298 expression in relation to viability on NTERA, NCI-H460 and THP-1 cells.

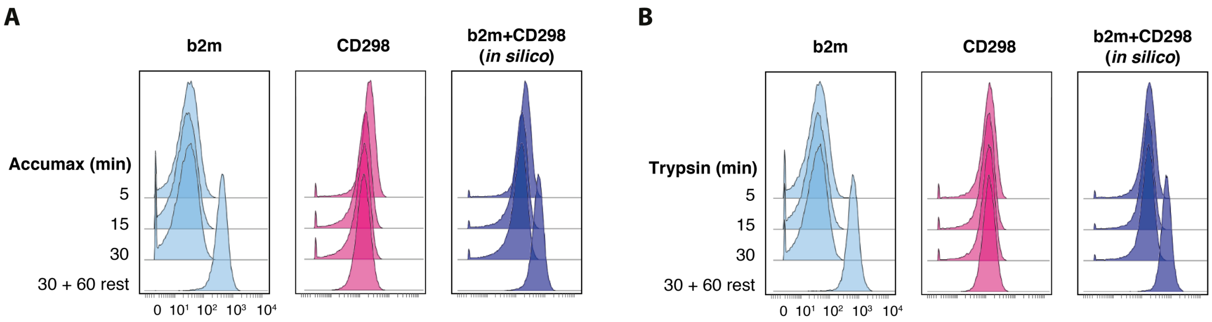

Fig. S2. Influence of digestive enzymes on b2m and CD298 expression. (A) NCI-H460 cells were incubated in Accumax solution for the indicated amount of time. Where stated, cells were washed after incubation, resuspended in cell culture medium and rested at 37 °C for 60 min. Shown is surface staining for b2m (198Pt, left) and CD298 (195Pt, middle). To assess combined signal from b2m and CD298 (right), measured single-cell intensities from both channels were added computationally. (B) NCI-H460 cells were treated with trypsin solution for the indicated amount of time. Shown is surface staining on live, single cells for b2m (198Pt, left) and CD298 (195Pt, middle) and combined signal (right).

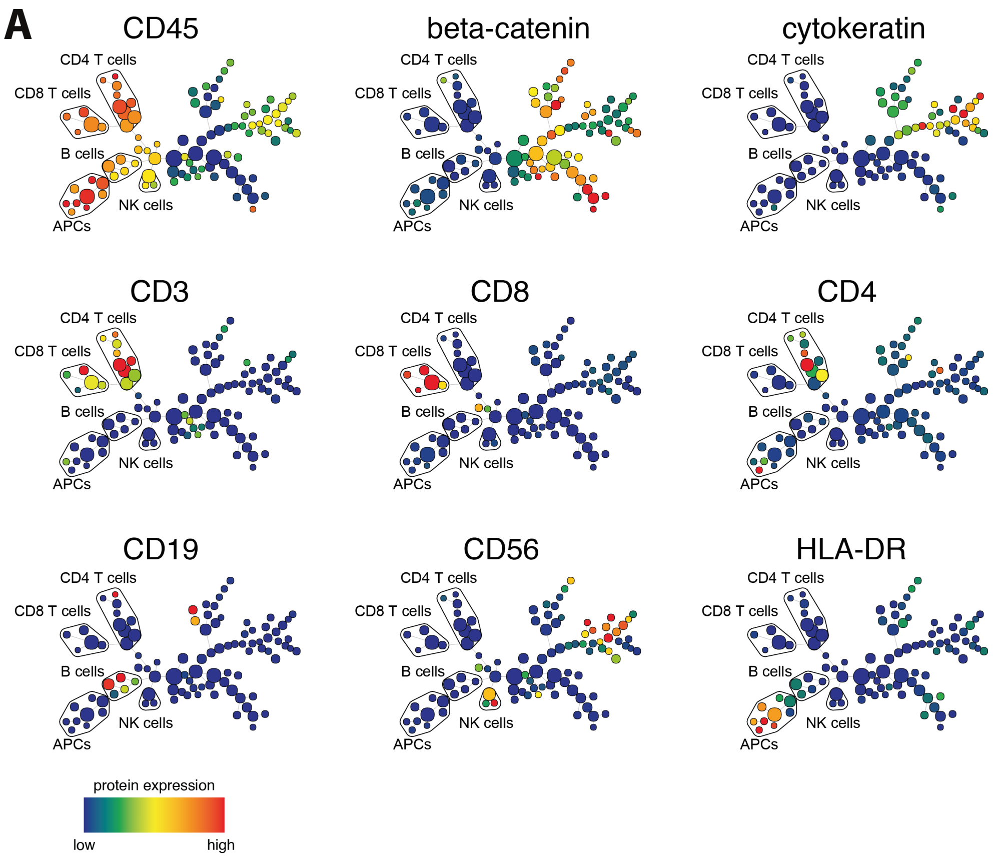

Fig. S3. SPADE representation of a tumor biopsy. (A) A tumor biopsy of a lung carcinoma patient was digested and prepared as a single-cell suspension. Cells were stimulated for up to 24 h *in vitro*, live cell barcoded and stained with heavy-metal conjugated antibodies (Table S1). The Cytobank implementation of SPADE was used to identify 100 clusters using the above displayed lineage-associated proteins.
